# Nonmedical Use of Antihistaminergic Anxiolytics and Other Prescription Drugs among Persons with Opioid Dependence

**DOI:** 10.1155/2016/9298571

**Published:** 2016-12-20

**Authors:** Disa Dahlman, Tove Abrahamsson, Alex H. Kral, Anders Hakansson

**Affiliations:** ^1^Department of Clinical Sciences Lund, Psychiatry, Faculty of Medicine, Lund University, Lund, Sweden; ^2^Malmo Addiction Centre, Malmo, Sweden; ^3^Behavioral and Urban Health Program, RTI International, San Francisco, CA, USA

## Abstract

*Background*. Nonmedical prescription drug use (NMPDU) is an increasing problem, insufficiently studied among people in opioid maintenance treatment (OMT). This study investigates the prevalence of and factors associated with NMPDU for drug classes insufficiently described in opioid-dependent populations, including antihistaminergic anxiolytics and central stimulants.* Methods*. Study participants were recruited at two OMT clinics in Malmo, Sweden, between October 2014 and December 2015 (*N* = 73) and interviewed about their use, motivations for use, and acquisition and administration of prescription drugs.* Results*. The majority of the sample reported lifetime NMPDU: 60% for benzodiazepine-like hypnotics (z-drugs), 21% for pregabalin, 19% for stimulants, and 12%–15% for antihistaminergic anxiolytics. Lower age was associated with nonmedical benzodiazepine use (Adjusted Odds Ratio = 0.89; 95% Confidence Interval = 0.82–0.97). Illicit acquisition was reported by 61% of people using z-drugs, 46% of people using pregabalin, and 38% of people using prescription stimulants, but only by 6–10% of people using antihistaminergic anxiolytics.* Conclusions*. The substantial nonmedical use of pregabalin, z-drugs, and prescription stimulants found in this study suggests that clinicians should prescribe these drugs with great caution. Nonmedical use of antihistaminergic anxiolytics does not seem to be a clinical issue among people in OMT in a Swedish setting, but we propose future studies to monitor their use.

## 1. Introduction

Nonmedical use of prescription drugs is a growing problem in many countries [1–3]. Previous research has shown that nonmedical use (use without a doctor's prescription, or in higher doses, more frequently, for longer duration or with another purpose than prescribed) of benzodiazepines is common among persons with opioid dependence, including both people who use illicit drugs and patients in opioid maintenance treatment (OMT) with methadone or buprenorphine [4–9]. Benzodiazepines can be used nonmedically by these individuals to potentiate the sedating effect of heroin and other opioids [6]. Prescription opioid analgesics can be used nonmedically together with or as a substitute for heroin or other strong opioids, since they are pharmacologically similar to heroin [10]. Furthermore, prescription drugs used to treat attention deficit/hyperactivity disorders (ADHD), including methylphenidate and other central stimulant medications, have a well-known abuse potential [11]. Nonmedical use of prescription stimulants is described in studies from the US [11–13] but is sparsely examined in non-US settings and among OMT patients specifically [14]. Frauger et al. [15] showed a rapid increase in methylphenidate use in France from 2005 to 2011 and noted an increased risk of nonmedical methylphenidate use among individuals with drug dependence.

In recent years, prescription sedatives and tranquilizers that were earlier considered nonaddictive are being investigated for their abuse potential, and nonmedical use has been reported among persons with heroin dependence. This is the case for the so-called z-drugs, benzodiazepine-like hypnotics including zolpidem, zaleplon, and zopiclone [16, 17], as well as the anxiolytic drug pregabalin [18]. In a study in Ireland, 23% of patients in methadone maintenance treatment (MMT) were using z-drugs nonmedically [16]. The percentage of nonmedical pregabalin use among patients in OMT has been estimated at 3–12% in previous studies [18–22].

Recent US studies have indicated that the prescription drug promethazine—an antihistamine which is often used to treat anxiety and sleep disorders (Sandoz Inc., 2006)—might also have a misuse potential among persons with opioid dependence [23], as well as among chronic pain patients [24] and in the general population [25]. The study by Shapiro et al. [23] investigated couse of promethazine and opioids and showed that 26% of patients in MMT had tested positive for promethazine, while only 15% of these had a valid prescription. We are not aware of any previous studies of the nonmedical use of other prescription antihistamines, such as alimemazine and hydroxyzine.

Promethazine can, alone or in combination with opioids, have negative health effects [23]. Promethazine can potentiate the sedating effect of opioids, increasing the risk for apnea and respiratory depression (Sandoz Inc., 2006). Promethazine prolongs cardiac repolarization time, which increases the risk of potentially lethal arrhythmias [26]. Overdose of promethazine is associated with delirium and neuroleptic malignant syndrome [27].

Apart from promethazine, nonmedical use of other prescription drugs is associated with several adverse health effects. Benzodiazepine use in combination with strong opioids is associated with overdose [6, 28, 29]. Z-drugs have sedative effects similar to those of benzodiazepines [30], and the risk of overdose and respiratory depression is increased if combining opioids and z-drugs [31]. Pregabalin may cause somnolence and confusion [32] and decrease the respiratory rate [33]. Concomitant opioid use is common in pregabalin-related fatalities, suggesting that the specific combination may increase overdose risk [34]. Nonoral administration of prescription drugs, for example, by crushing and snorting or injecting, may generally cause tissue damage and vein damage and increase the risk of infections and thrombosis [35–38].

The aim of the current study was to describe the prevalence of use and nonmedical use, correlates of nonmedical use, motivations for use, and acquisition and administration of prescription drugs for drug classes insufficiently described in opioid-dependent populations, including antihistaminergic and other anxiolytics (promethazine, alimemazine, hydroxyzine, z-drugs, and pregabalin) and central stimulants.

## 2. Methods

### 2.1. Study Population

The sample consisted of 73 persons with opioid dependence currently in OMT with buprenorphine, buprenorphine-naloxone, or methadone. Recruitment of respondents took place at two OMT clinics in Malmo, Sweden, between October 2014 and December 2015. Respondents were chosen randomly. The only inclusion criterion for participation in the study was current OMT. Exclusion criteria were inability to understand the information or complete the interview (e.g., due to language difficulties or severe psychiatric symptoms).

All recruitment steps of participants and all interviews were conducted by one nurse and one assisting nurse, both experienced in the care of patients with substance use disorders. Before each interview, the respondent received oral and written information about the study and gave written consent to participation. Study participants received a gift card valid for SEK 100 (USD $12) for completing the questionnaire. The study was approved by the Regional Ethics Board in Lund, Sweden (file number 2013/877).

### 2.2. Instruments and Measures

The study was based on self-reports from structured interviews. The interviews were performed according to a survey based on two questionnaires developed at RTI International with the purpose of investigating nonmedical use of promethazine [23] and general nonmedical use of prescription drugs, respectively. The questions were translated to Swedish and in a few cases adjusted to Swedish conditions. Some new questions were added, concerning duration of OMT, use of illicit drugs during periods of active drug use, and experienced effects of combining heroin or OMT drugs (i.e., methadone or buprenorphine) with the prescription drug in question.

Prescription antihistamines including promethazine, alimemazine, and hydroxyzine were investigated in the survey. We also included prescription drugs that have a known, but insufficiently documented, abuse potential: the benzodiazepine-like hypnotics zopiclone, zolpidem, and zaleplon and the anxiolytic drug pregabalin [39]. We also included methylphenidate and other prescription stimulants in the questionnaire, since nonmedical use of these prescription drugs is poorly described in the Swedish population [14]. Prescription drugs with well-established abuse potential (benzodiazepines and opioid analgesics) were also included in the questionnaire as reference substances. Pregabalin, z-drugs, promethazine, hydroxyzine, and alimemazine were included in the questionnaire with generic name as well as Swedish brand names. A list of examples (substance and/or brand names) was provided for benzodiazepines (oxazepam [Sobril®], diazepam [Stesolid®/Valium®], clonazepam [Iktorivil®], alprazolam [Xanor®], flunitrazepam [Rohypnol®], lorazepam [Temesta®], nitrazepam [Apodorm®], bromazepam, phenazepam, and temazepam), prescription opioids (codeine [Citodon®/Treo Comp®/Paraflex Comp®], ketobemidone [Ketogan®], tramadol [Tiparol®/Nobligan®], fentanyl [Durogesic®, etc.], morphine, Dolcontin®, hydromorphone, oxycodone [OxyContin®/OxyNorm®], dextropropoxyphene [Dexofen®/Doloxene®]), and prescription stimulants (methylphenidate, amphetamines [Concerta®, Ritalin®, Medikinet®, Equasym®, Adderall®, and Metamina®]). The nonaddictive ADHD medication atomoxetine (Strattera®) was explicitly excluded from prescription stimulants in the questionnaire.

The same 10 questions were asked for all prescription drugs. Lifetime use, lifetime nonmedical use, lifetime approach by someone trying to sell prescription drugs, lifetime use in combination with heroin, methadone, or buprenorphine (assessed separately) were* yes/no* questions. Lifetime nonmedical use was assessed through the question (translated from Swedish) “Have you ever used [prescription drug] without a prescription or in another way than prescribed (such as more frequently, in higher dose or for another reason than prescribed)?”

The question regarding current prescription had three choices (*no/yes/no, but previously*). Those who reported combined use of either of the prescription drugs and heroin and methadone or buprenorphine/buprenorphine-naloxone were asked to answer the yes/no question “Have you experienced any special effect from combining the drugs, compared to when you have used them separately?” A positive answer was followed by space for describing the effect (“Describe the effect in your own words.”).

Four questions were multiple choice questions. Usual ways of acquiring each prescription drug had the choices* prescription/bought from the black market/other (specify)*. Motives for current or previous use of prescription drugs and current or previous combination of prescription drugs and strong opioids had the choices* get high/relieve physical problems for example, pain/relieve emotional problems for example*, and* anxiety/other (specify)*. Lifetime ways of administration had the choices* swallowed/snorted/injected/smoked/other (specify)*.

### 2.3. Statistical Analysis

In bivariate and multivariate analysis, the outcome variables were self-reported lifetime nonmedical use of each of the prescription drugs assessed in the questionnaire. The independent variables were sex (dichotomized into male versus female/not defined), age (continuous), and type of OMT medication (buprenorphine versus methadone). We used Chi square test to assess binary variables and Mann–Whitney test for the continuous variable. In multivariate analyses, we included all three independent variables regardless of significance in bivariate analysis. In order to avoid an overinclusion of variables in analyses of outcomes with a low absolute number of positive cases, the number of potential predictors in regression analyses was set to correspond to five cases per variable [40]. Missing values were excluded from the analyses. *p* values below 0.05 were considered statistically significant. All statistical analyses were performed in SPSS (version 21) [41].

## 3. Results

### 3.1. Population Characteristics

Female participants constituted 30% of the sample and the median age was 43 years (range 22–66 years) (Table 1). Fifty-eight percent of patients received OMT with methadone and 37% with buprenorphine or buprenorphine-naloxone. Heroin, benzodiazepines, and cannabis were the most commonly used drugs during the 30 days prior to OMT start.

### 3.2. Use and Nonmedical Use of Prescription Drugs

Lifetime nonmedical use was reported for all prescription drugs, with the highest prevalence for drugs with well-established addictive potential: 81% for benzodiazepines, 67% for prescription opioids, 60% for z-drugs, 21% for pregabalin, 19% for prescription stimulants, 12% for promethazine, 12% for hydroxyzine, and 15% for alimemazine (Table 2).

In multivariate logistic regression analysis, lower age was significantly associated with reported nonmedical use of benzodiazepines (Table 3). Associations between lower age and nonmedical use of pregabalin did not reach statistical significance.

A majority of people using prescription drugs reported oral consumption only (Table 4). A small number of people using benzodiazepines, z-drugs, and prescription opioids reported administration through snorting and smoking. Injection was reported by 16% of people using benzodiazepines, 2% of people using z-drugs, 22% of people using prescription opioids, and 13% of people using prescription stimulants.

### 3.3. Motivation for Use of Prescription Drugs

When including only those who reported lifetime use of each prescription drug, no participants who used promethazine reported having used for the purpose of getting high, and only 4% of participants who used hydroxyzine or alimemazine reported this as a reason for using the drug (medically and nonmedically). However, for z-drugs, pregabalin, prescription stimulants, benzodiazepines, and prescription opioids, 23–35% of participants reported use with the purpose of getting high (Table 5). The most common motivations for promethazine, hydroxyzine, and alimemazine use were “relief of emotional problems” and “other purposes.” “Other purposes” included improved sleep and calming effects. No persons using antihistamines reported motivations related to drug improvement or influence.

A substantial percentage of individuals who combined prescription drugs with heroin, methadone, or buprenorphine reported additional effects from combined use. This was reported by 71% for benzodiazepines, 67% for z-drugs, 65% for pregabalin, and 61% for prescription opioids and was also prevalent for prescription stimulants (39%), promethazine (32%), alimemazine (30%), and hydroxyzine (20%). Specifications of these additional effects included drug potentiating effects such as “increased drug effect” (benzodiazepines, pregabalin, z-drugs, alimemazine, prescription opioids, and prescription stimulants), “doubled effect” (promethazine), and “pleasant” (benzodiazepines, pregabalin, and z-drugs).

### 3.4. Acquisition of Prescription Drugs

Illicit acquisition from the black market was common among people who had used z-drugs (61%), pregabalin (46%), and prescription stimulants (36%) but only reported by 6–10% of people who had used antihistaminergic anxiolytics (Table 6). In comparison, the percentage reporting that they typically acquired benzodiazepines and prescription opioids from the black market was 81 and 72%, respectively.

For all prescription drugs, there were subjects reporting that they had been approached by someone trying to sell the drug. This was most common for benzodiazepines (78%), prescription opioids (71%), and z-drugs (70%), less common for pregabalin (43%) and prescription stimulants (32%), and least common for promethazine, hydroxyzine, and alimemazine (range 8–10%).

## 4. Discussion

This study provides new data on nonmedical use of a number of prescription drugs among individuals with opioid dependence, a subject which has previously not been comprehensively studied outside the US [1, 42]. We found that nonmedical use of pregabalin, z-drugs, and prescription stimulants was highly prevalent, for recreational as well as self-treating purposes. While lifetime use of prescription antihistamines including promethazine was highly prevalent, self-reported nonmedical use of these drugs was not common.

In this study, common purposes for the use of prescription antihistamines both separately and in combination with strong opioids were relief of emotional problems and improved sleep, in line with the intended purposes for medical use. Illicit acquisition of prescription antihistamines was uncommon (6–10%), and less than one in ten of the sample had been approached by someone who tried to sell promethazine, hydroxyzine, or alimemazine, respectively. Still, 12% reported nonmedical use of hydroxyzine and alimemazine, and a small number of subjects who had combined strong opioids with antihistamines reported a “better effect” or additional effects such as “weird feeling.”

z-drugs and pregabalin are considered addictive in clinical practice, but research is sparse. This study strongly supported the misuse potential of z-drugs among people with opioid dependence [16, 17] with almost three-quarters of people who reported z-drug lifetime use also reporting nonmedical use. It is also notable that a quarter of those who reported lifetime pregabalin use used it for recreational purposes. The percentage was the same among those who reported lifetime use of benzodiazepines, which have a well-known addictive and misuse potential [6–8]. Reported lifetime nonmedical use of pregabalin was 21%, compared to the previously reported 3–12% point prevalence among patients in OMT [18–22], 17% in urinary samples from Swedish patients in OMT [14], and 0.5% among the general UK population [43]. Substitution for benzodiazepines was a specific purpose for pregabalin use. Also worth noting is that 35% of people who reported combined use of pregabalin and opioids reported “get high” as a reason for combining them.

A majority of study participants had been approached by someone trying to sell z-drugs, and illicit trade was the most common way of obtaining z-drugs. For pregabalin, half of those who reported lifetime pregabalin use reported illicit obtaining. This finding is similar to previous research from the UK, where 58% of people misusing pregabalin reported acquisition from family or acquaintances and 47% from the Internet [43]. The pattern of z-drug as well as pregabalin use suggests that there is an illicit market for z-drug and pregabalin trade in Sweden.

While there have been reports of nonmedical use of promethazine in combination with opioids from the US [23, 24], India [44], Vietnam [45], and Nepal [46], this, as well as nonmedical use of other prescription antihistamines (hydroxyzine and alimemazine), was uncommon in the present study, in combination with opioids or by itself. However, lifetime medical use of prescription antihistamines was high, reflecting that these drugs are commonly prescribed in Swedish medical care. Reasons for these regional differences are difficult to understand; however they are coherent with previous studies on other substances. Usage patterns of both illicit and prescription drugs have been shown to differ greatly between European countries [47], as well as within countries such as Switzerland, Germany [48], and the US [49].

Nonmedical use of prescription stimulants rarely has been assessed specifically in OMT patients. From this study, nonmedical use appears to be a significant issue. Interestingly, in the present study, 29% of those who reported prescription stimulant use reported that they used these drugs to “get high,” while no responders reported “relieve emotional problems” as a purpose for use. Three subjects reported injection use of prescription stimulants. Nonmedical use of prescription stimulants is described in various subpopulations such as high school and college students in the US [11, 50, 51] and also among people with substance disorders [15, 52]. Our study indicates that prescription stimulants have a substantial abuse potential among people with opioid dependence. This is in line with a Swedish study [14] detecting methylphenidate in urine samples from 23% of patients in OMT, of which only a small minority had a valid prescription. Patients in OMT may also use prescription stimulants in order to self-treat ADHD symptoms. Previous research has shown that ADHD is highly prevalent among adults with substance use disorders [53, 54].

The results from this study implicate that there is substantial nonmedical use and illicit trade of pregabalin, z-drugs, and prescription stimulants. Pregabalin [19, 55] and z-drugs [17] have been introduced as better substitutes for benzodiazepines and considered to have less addiction and misuse potential. One hypothesis is that drugs that do not have any abuse or dependence potential in the general population might still be used nonmedically by people with opioid dependence, possibly due to effects from combining these drugs with heroin, methadone, or buprenorphine.

The only characteristic independently associated with nonmedical prescription drug use was higher odds for reported nonmedical use of benzodiazepines among younger subjects in the study. The association between younger age and nonmedical use of pregabalin did not reach statistical significance, possibly due to a small number of participants. This is in accordance with several previous studies, which have found an association between nonmedical use of prescription drugs and younger age [56–58].

This study has some potential limitations. First, the number of participants was small, and the participants were recruited from two OMT clinics in one city in southern Sweden. More studies are therefore necessary to examine whether our findings are applicable in other settings. Due to the relatively small number of participants, we might have missed possible predictors of nonmedical use or purposes for use. With a larger study sample, it would have been particularly interesting to investigate whether use of street drugs or alcohol or comorbid psychiatric disorders is associated with nonmedical prescription drug use. In addition, illicit drug use data and alcohol data were not available for the lifetime period for which the outcome variables of the present study were assessed; thus, although Swedish OMT regulations require the opioid dependence to be the predominant drug use pattern of individuals entering OMT [9], the lack of systematic data for other street drugs is a study limitation. We did, however, not have any reason to suspect that the sample was not representative for patients at the OMT clinics. Age and gender distribution were similar to previous Swedish studies on patients in OMT [59, 60]. Use of street drugs and benzodiazepines was comparable to other clinical studies on patients in OMT in the southern Swedish region [9].

Second, the study is based on self-reports, which might be subject to recall bias as well as incorrect information/underreporting due to being in a rush, or because of fear of consequences for their treatment at the OMT clinic where the interviews took place. Some persons invited to participate in the study declined with the motivation that they did not want to share information of previous substance misuse. To minimize the latter kind of risk, all participants were given explicit information about the confidential status of their replies and that the interview contained no questions specifically regarding nonmedical substance use during OMT. However, there is a possibility that nonmedical use and illicit acquisition is underreported.

The results from this study have clinical implications. Since several of the prescription drugs assessed are commonly used and prescribed in clinical practice, their potential for nonmedical use, attractiveness on the drug market, and ways of administration are of clinical concern. In the light of the results from this study, we suggest caution when prescribing pregabalin, z-drugs, and prescription stimulants to persons with opioid dependence.

In conclusion, nonmedical use of antihistaminergic anxiolytic does not seem to be a clinical issue among people with opioid dependence in a Swedish setting, while there is substantial nonmedical use of pregabalin, z-drugs, and prescription stimulants. Even though the interest in prescription antihistamines for recreational purposes seems weak in the current study, we suggest future studies monitoring the prevalence of nonmedical use as well as qualitative studies assessing motivations for use and combination with strong opioids. More studies are needed to assess the extent and motivations of nonmedical prescription drug use among people with opioid dependence.

## Figures and Tables

**Table 1:** Sample characteristics among opioid maintenance treatment patients in Malmo, Sweden (*N* = 73).

Characteristic	*n* (%)	Median years (range)
*Sex*		
Male	47 (64%)	
Female	22 (30%)	
Transgender/do not wish to define	4 (6%)	
*Age in years *		43 (22–66)
*OMT medication*		
Methadone	42 (58%)	
Buprenorphine or buprenorphine-naloxone	27 (37%)	
*Missing*	*4 (5%)*	
*Years in OMT *		2 (0–8)
*Missing n* = 8		
*Illicit drug use in the last 30 days before OMT start*		
Heroin	63 (86%)	
Cannabis	29 (40%)	
Cocaine	23 (32%)	
Amphetamine	18 (25%)	
*Nonmedical prescription drug use in the last 30 days before OMT start*		
Benzodiazepines	41 (56%)	
Methadone	28 (38%)	
Other prescription opioids	28 (38%)	
Buprenorphine	18 (25%)	
Prescription stimulants	10 (14%)	

**Table 2:** Use and non-medical use of prescription drugs (*N* = 73).

Prescription drug	Ever used^a^	Ever used non-medically^b^	Ever used in combination with heroin, methadone, or buprenorphine/ Suboxone^c^
Benzodiazepines	62 (85%)	59 (81%)	59 (81%)
Pregabalin	26 (36%)	15 (21%)	17 (23%)
z-drugs	62 (85%)	44 (60%)	46 (63%)
Promethazine	50 (69%)	9 (12%)	25 (34%)
Hydroxyzine	45 (62%)	9 (12%)	20 (27%)
Alimemazine	52 (71%)	11 (15%)	30 (41%)
Prescription opioids	60 (82%)	49 (67%)	41 (56%)
Prescription stimulants	24 (33%)	14 (19%)	13 (18%)

^a^Missing values *n* = 1 for pregabalin and promethazine; *n* = 2 for hydroxyzine and alimemazine.

^b^Missing values *n* = 1 for z-drugs; *n* = 2 for alimemazine; and *n* = 4 for promethazine and hydroxyzine.

^c^Missing values *n* = 1 for pregabalin; *n* = 2 for prescription stimulants; *n* = 3 for z-drugs; *n* = 4 for prescription opioids; *n* = 7 for alimemazine; and *n* = 8 for promethazine and hydroxyzine.

**Table 3:** Factors associated with nonmedical use of prescription drugs. Multivariable logistic regression analysis.

Characteristic	Benzodiazepines^a^	Pregabalin^b^	z-drugs^c^	Promethazine/ hydroxyzine/ alimemazine^d^	Prescription opioids^e^	Prescription stimulants^f^
	AOR (95% CI)	AOR (95% CI)	AOR (95% CI)	AOR (95% CI)	AOR (95% CI)	AOR (95% CI)
*Gender *	0.69 (0.15–3.09)	0.31 (0.08–1.31)	0.60 (0.20–1.79)	0.88 (0.28–2.79)	0.47 (0.14–1.56)	0.67 (0.15–2.20)
(Male versus female/nonbinary)						

*OMT type*	2.76 (0.61–12.49)	0.46 (0.12–1.78)	0.68 (0.22–2.08)	0.90 (0.28–2.89)	1.13 (0.34–3.79)	**0.26 ** **(0.07**–1.01)^+^
(Buprenorphine/buprenorphine-naloxone versus methadone)						

*Age *	**0.89 **	**0.94 ** **(0.88**–1.00)^+^	1.00 (0.95–1.06)	1.02 (0.96–1.08)	0.95 (0.90–1.01)	0.98 (0.92–1.05)
(Years, continuous)	**(0.82**–0.97)^**∗**^					

^*∗*^ *p* < 0.05.

^+^ *p* < 0.10.

^a^Missing values *n* = 4; *n* = 69 included in analysis.

^b^Missing values *n* = 5; *n* = 68 included in analysis.

^c^Missing values *n* = 5; *n* = 68 included in analysis.

^d^Computed variable (missing promethazine *n* = 5, hydroxyzine *n* = 6, and alimemazine *n* = 4); missing values *n* = 4; *n* = 69 included in analysis.

^e^Missing values *n* = 4; *n* = 69 included in analysis.

^f^Missing values *n* = 4; *n* = 69 included in analysis.

**Table 4:** Administration of prescription drugs (not mutually exclusive) among opioid maintenance treatment patients. Presented as *n* (% of lifetime users of each prescription drug).

Route of administration	Benzodiazepines^a^	Pregabalin	z-drugs	Promethazine^b^	Hydroxyzine^c^	Alimemazine^c^	Prescription opioids^a^	Prescription stimulants^d^
Oral consumption	59/62 (95%)	26/26 (100%)	61/62 (98%)	45/50 (90%)	42/45 (93%)	49/52 (94%)	55/60 (92%)	19/24 (79%)
Snorting	2/62 (3%)	0	1/62 (2%)	0	0	0	4/60 (7%)	0
Injection	10/62 (16%)	0	1/62 (2%)	0	0	0	13/60 (22%)	3/24 (13%)
Smoking	5/62 (8%)	0	0	0	0	0	6/60 (10%)	0
Other routes	0	0	1/62 (2%); *not specified*	0	0	0	1/60 (2%); *mixed with tea*	1/24 (4%); *chewed*

^a^Missing *n* = 1.

^b^Missing *n* = 5.

^c^Missing *n* = 3.

^d^Missing  *n* = 2.

**Table 5:** Motivation for use of prescription drugs in general and in combination with strong opioids (heroin, methadone, and buprenorphine). Not mutually exclusive. Presented as *n* (% of persons reporting lifetime use/combined use).

Characteristic	Benzodiazepines	Pregabalin	z-drugs	Promethazine	Hydroxyzine	Alimemazine	Prescription opioids	Prescription stimulants
*Motives for use of prescription drugs* ^a^								
Recreational use (“get high”)	16/62 (26%)	7/26 (27%)	14/62 (23%)	0	2/45 (4%)	2/52 (4%)	21/60 (35%)	7/24 (29%)
Relieve physical problems	12/62 (19%)	3/26 (12%)	2/62 (3%)	0	0	1/52 (2%)	29/60 (48%)	1/24 (4%)
Relieve emotional problems	43/62 (69%)	17/26 (65%)	18/62 (29%)	22/50 (44%)	24/45 (53%)	20/52 (39%)	11/60 (18%)	0
Other motives	17/62 (27%)	5/26 (19%)	37/62 (60%)	25/50 (50%)	16/45 (36%)	28/52 (54%)	18/60 (30%)	14/24 (58%)

*Additional effect from combining prescription drugs with* *opioids*^b^	42/59 (71%)	11/17 (65%)	31/46 (67%)	8/25 (32%)	4/20 (20%)	9/30 (30%)	25/41 (61%)	5/13 (39%)

^a^Missing values *n* = 3 for prescription stimulants; *n* = 4 for alimemazine; *n* = 6 for promethazine; and *n* = 7 for hydroxyzine.

^b^Missing values *n* = 1 for pregabalin; *n* = 3 for promethazine, alimemazine, and prescription stimulants; *n* = 4 for z-drugs; *n* = 5 for benzodiazepines and hydroxyzine; and *n* = 8 for prescription opioids.

**Table 6:** Usual ways of acquiring prescription drugs (not mutually exclusive) among people reporting lifetime use of each substance, respectively. *n* (% of lifetime users/% of study sample).

	Benzodiazepines	Pregabalin	z-drugs	Promethazine	Hydroxyzine	Alimemazine	Prescription opioids	Prescription stimulants
Current prescription^a^	8/62 (13%)	5/26 (19%)	12/62 (19%)	22/50 (44%)	12/45 (27%)	12/52 (23%)	12/60 (20%)	7/24 (29%)
Usual acquisition through prescription^b^	13/62 (21%)	9/26 (35%)	23/62 (37%)	32/50 (64%)	29/45 (64%)	34/52 (65%)	19/60 (32%)	12/24 (50%)
Usual acquisition from black market^b^	50/62 (81%)	12/26 (46%)	38/62 (61%)	3/50 (6%)	3/45 (7%)	5/52 (10%)	43/60 (72%)	9/24 (38%)
Other usual acquisition^b^	6/62 (10%)	4/26 (15%)	7/62 (11%)	12/50 (24%)	7/45 (16%)	9/52 (17%)	1/60 (2%)	4/24 (17%)
Gift	2	4	3	3	1	3	1	3
In treatment	1	—	3	9	6	5	—	—
Facility/prison								

Ever approached by someone trying to sell prescription drug^c^	57 (78%)	31 (43%)	51 (70%)	6 (8%)	7 (10%)	6 (8%)	52 (71%)	23 (32%)
* n* (% of 73)								

^a^Missing values *n* = 1 for benzodiazepines and prescription stimulants; *n* = 2 for hydroxyzine and prescription opioids; *n* = 3 for alimemazine; and *n* = 5 for promethazine.

^b^Missing values *n* = 1 for pregabalin and prescription stimulants; *n* = 2 for benzodiazepines; *n* = 3 for z-drugs and promethazine; *n* = 4 for alimemazine and prescription opioids; and *n* = 7 for hydroxyzine.

^c^Missing values *n* = 1 for z-drugs; *n* = 2 for hydroxyzine, alimemazine, prescription opioids, and prescription stimulants; *n* = 3 for promethazine; and *n* = 5 for benzodiazepines.
